# Mobile colistin resistance (MCR), extended-spectrum beta-lactamase (ESBL) and multidrug resistance monitoring in *Escherichia coli* (commensal and pathogenic) in pig farming: need of harmonized guidelines and clinical breakpoints

**DOI:** 10.3389/fmicb.2022.1042612

**Published:** 2022-12-02

**Authors:** Vanesa García, Isidro García-Meniño, Verónica Gómez, Miguel Jiménez-Orellana, Antonio Méndez, Alvaro Aguarón, Elisabet Roca, Azucena Mora

**Affiliations:** ^1^Dpto. de Microbioloxía e Parasitoloxía, Laboratorio de Referencia de Escherichia coli (LREC), Universidade de Santiago de Compostela (USC), Lugo, Spain; ^2^Instituto de Investigación Sanitaria de Santiago de Compostela (IDIS), Santiago, Spain; ^3^Consellería do Medio Rural, Laboratorio de Sanidade e Produción Animal de Galicia (LASAPAGA), Subdirección xeral de Gandaría. Dirección Xeral de Gandaría, Agricultura e Industrias Agroalimentarias, Lugo, Spain; ^4^Laboratorios Syva S.A. Servicios Técnicos de Porcino, Parque Tecnológico de León, León, Spain

**Keywords:** *Escherichia coli*, MCR, ESBL, colistin, fluoroquinolones, swine, multidrug resistance, EUCAST

## Abstract

Current data on antimicrobial resistance in pig production is essential for the follow-up strategic programs to eventually preserve the effectiveness of last-resort antibiotics for humans. Here, we characterized 106 *Escherichia coli* recovered in routine diagnosis (2020–2022) from fecal sample pigs, belonging to 74 Spanish industrial farms, affected by diarrhea. The analysis of virulence-gene targets associated with pathotypes of *E. coli*, determined 64 as pathogenic and 42 as commensal. Antimicrobial susceptibility testing (AST) performed by minimal inhibitory concentration (MIC) assay, was interpreted by applying breakpoints/cut-off values from the different standards EUCAST/TECOFF 2022, CLSI VET ED5:2020, and CASFM VET2020. Comparisons taking EUCAST as reference exhibited moderate to high correlation except for enrofloxacin, neomycin, and florfenicol. Of note, is the lack of clinical breakpoints for antibiotics of common use in veterinary medicine such as cefquinome, marbofloxacin, or florfenicol. AST results determined multidrug resistance (MDR) to ≥3 antimicrobial categories for 78.3% of the collection, without significant differences in commensal *vs* pathogenic isolates. Plasmid-mediated mobile colistin resistance gene (*mcr*) was present in 11.3% of 106 isolates, all of them pathogenic. This means a significant decrease compared to our previous data. Furthermore, 21.7% of the 106 *E. coli* were ESBL-producers, without differences between commensal and pathogenic isolates, and *mcr*/ESBL genes co-occurred in 3 isolates. Phylogenetic characterization showed a similar population structure (A, B1, C, D, and E), in both commensal and pathogenic *E. coli*, but with significant differences for B1, C, and E (38.1 *vs* 20.3%; 19 *vs* 1.6%; and 7.1 *vs* 25%, respectively). Additionally, we identified one B2 isolate of clone O4:H5-B2-ST12 (CH13-223), positive for the uropathogenic (UPEC) status, and *in silico* predicted as human pathogen. We suggest that a diagnosis workflow based on AST, detection of *mcr* and ESBL genes, and phylogenetic characterization, would be a useful monitoring tool under a “One-Health” perspective.

## Introduction

Currently, antimicrobial stewardship programs are being implemented in all environments under a “One Health” perspective, including hospital, community, and agricultural settings (McEwen and Collignon, 2018). Following the European Medicines Agency’s (EMA) advice on the use of antibiotics, the Spanish Medicines and Healthcare Products Agency (AEMPS) launched in 2014 the National Plan against Antibiotic Resistance (PRAN) as a strategic action plan to reduce the risk of selection and dissemination of antibiotic resistance and, consequently, reduce the impact on human and animals’ health, sustainably preserving the effectiveness of existing antibiotics. At the end of 2016, representatives of the national associations of veterinarians and professionals from the pig production sector signed, together with PRAN, the agreement for the voluntary Reduction of Colistin Consumption in the Pig Farming Sector (“REDUCE” program). The main aim of this alliance was to reduce the consumption of colistin to 5 mg/population correction unit (PCU) in 3 years and control the alternative consumption of neomycin and/or apramycin. Six years after the beginning of the program, the result is a reduction of almost 100% in the use of colistin in this sector, from 52 to 0.4 mg/PCU (European Medicines Agency, 2021).

Swine colibacillosis is one of the major challenges for the pig industry worldwide due to the high morbidity and mortality rates, and derived costs from prevention and antimicrobial treatment. This multifactorial syndrome caused by *Escherichia coli*, exhibits three main disease conditions (edema disease, neonatal and post-weaning diarrhea), which can be differentiated by the age range of the affected animals, pathogenesis, and *E. coli* pathotype. Among all, enterotoxigenic *E. coli* (ETEC) is the most prevalent pathotype, together with Shiga toxin–producing *E. coli* (STEC) and atypical enteropathogenic *E. coli* (aEPEC). Hybrid strains (ETEC/STEC) are also relatively common (Luppi, 2017; García-Meniño et al., 2018).

Our previous studies on antibiotic resistance in Spanish pig farming (2005–2017), revealed a high prevalence of colistin resistance (76.9%), associated with the presence of plasmidic *mcr* genes (mainly *mcr-4*, but also *mcr-1* and *mcr-5*), and multidrug resistance (MDR; > 85%) within 186 ETEC, STEC and ETEC/STEC isolates implicated in post-weaning diarrhea (PWD) (García-Meniño et al., 2021). Remarkably, the potentially zoonotic of the extraintestinal pathogenic *E. coli* (ExPEC) lineage B2-ST131 (García-Meniño et al., 2018) was identified in 3.6% of 499 *E. coli* (18 ST131 isolates, including 7 *mcr* carriers) from diarrheal pigs, some of them showing genetic and genomic similarities with human clinical isolates (García-Meniño et al., 2018; Flament-Simon et al., 2020a).

We are currently facing big challenges in the use of antibiotics in veterinary medicine. On the one hand, in January 2022 the new legislation on veterinary medicines [Regulation (EU) 2019/6; Reglamento (UE), 2019] and medicated feed [Regulation (EU) 2019/4; Reglamento (UE), 2019] came into force, which, among others, limits the use of antibiotic prophylaxis and metaphylaxis. Besides, the EMA recently proposed a new categorization of antimicrobials, due to the risk of resistance development associated with use in animals and the potential impact on humans. This proposal establishes four categories, with Category A (“Avoid”) including those antimicrobials not currently authorized in veterinary medicine in the European Union, such as fosfomycin or monobactams; and with Category B (“Restrict”) including those restricted in animals to mitigate the risk to public health, namely, quinolones, broad-spectrum cephalosporins and polymyxins (EMA/688114/, 2020).

In the present study, we characterized a collection of 106 *E. coli* recovered in routine diagnosis (2020–2022) from fecal sample pigs, belonging to 74 Spanish industrial farms, affected by diarrhea, with two main aims: (i) to gain knowledge on the status of antimicrobial resistance in Spanish swine production; (ii) to analyze diagnosis and clinical difficulties in the veterinary sector when providing the best antibiotic therapy option in food production pigs.

## Materials and methods

### Samples and *Escherichia coli* collection

The study collection analyzed here, consisted of 106 swine *E. coli* isolates recovered from 93 stool samples of pigs reared on 74 farms, managed by 35 different pig companies. The farms involved here, belong to 19 Spanish provinces of those Autonomous Communities (10 out of 17) with the highest census of pig livestock in 2020, according to data from the Ministry of Agriculture, Fisheries and Food (Government of Spain): Aragon, Cataluña, Castilla y León, Andalucía, Región de Murcia, Extremadura, Castilla-La Mancha, Galicia, Comunidad Valenciana, Comunidad Foral de Navarra. Three production stage groups were included: lactation, transition, and fattening (38, 49 and 6 samples, respectively). The samples, either fecal swabs or isolation plates of MacConkey agar (LMAC, Oxoid), were received at the Spanish Reference Laboratory of *E. coli* (LREC, USC), and tested for routine diagnosis of enteric colibacillosis between September 2020 and April 2022. A brief voluntary questionnaire was performed about the use of specific drugs (ceftiofur, enrofloxacin/marbofloxacin, neomycin, colistin, sulfonamides, florfenicol, tiamulin) for infectious treatment in the farms involved in this study.

Briefly, the confluent growth from the fecal swabs plated on lactose MacConkey agar (LMAC, Oxoid), and incubated at 37°C for 18–24 h, was subjected to polymerase chain reaction (PCR) to detect the presence of ETEC, STEC, and EPEC using specific genetic targets encoding toxins (LT, STa, STb, and Stx2e), intimin (Eae) and fimbriae (F4, F5, F6, F18, and F41) as previously detailed (García-Meniño et al., 2021) (Supplementary Table S1). Additionally, confluents were also screened for *rbf*O25 associated with the pandemic lineage B2-ST131 (Clermont et al., 2008) (Supplementary Table S2). From PCR-positive confluents, different *E. coli*-like colonies (up to 10) were selected and plated on tryptone soy agar (TSA, Oxoid), which were in turn individually analyzed by PCR. Per sample, all *E. coli* colonies with different virulence-gene profiles were stored at room temperature in nutrient broth (Difco) with 0.75% nutrient agar (Difco) for further characterization. Likewise, from plates with negative confluents for all virulence targets, one *E. coli*-like colony was tested to confirm the status of non-pathogenic *E. coli* and conserved. PCR amplification of the β-d-glucuronidase-encoding gene (*uidA*) was routinely used to confirm the identity of the species *E. coli* (Gómez-Duarte et al., 2010) (Supplementary Table S2), which was further confirmed at the Laboratorio de Sanidade Animal de Galicia (LASAPAGA, Lugo, Spain) by matrix-assisted laser desorption/ionization–time-of-flight mass spectrometry (MALDI-TOF; Bruker Daltonik, Bremen, Germany) after conventional culture. The phylogroup of the isolates was established according to the PCR-based method developed by Clermont et al. (2013, 2019) (Supplementary Table S3), which recognizes eight phylogroups belonging to *E. coli sensu stricto* (A, B1, B2, C, D, E, F, and G).

### Antimicrobial susceptibility testing

Minimal inhibitory concentrations (MICs) of the 106 *E. coli* isolates were determined at the LASAPAGA using the VITEK^®^ 2 (bioMérieux, Inc., Hazelwood, MO, United States) systems for conventional AST (bioMérieux, Spain) with AST-GN96 test kit cards, following the manufacturer’s recommendations. The following drugs and categories were included in the analysis: aminopenicillins (ampicillin); aminopenicillins in combination with beta-lactamase inhibitor (amoxicillin/clavulanic acid, ticarcillin/clavulanic acid); non-broad spectrum cephalosporins (cefalexin and cefalotin); broad-spectrum cephalosporins (cefoperazone, ceftiofur, and cefquinome); carbapenems (imipenem); fluoroquinolones (enrofloxacin, flumequine, and marbofloxacin); aminoglycosides (gentamicin and neomycin); tetracyclines (tetracycline); polymyxins (polymyxin B); sulfonamides, dihydrofolate reductase inhibitors, and combinations (trimethoprim, trimethoprim/sulphamethoxazole); amphenicoles (florfenicol). The florfenicol resistance was double tested for a representative group of 42 isolates using MIC and disk diffusion assays. In addition, and due to our previous data of high prevalence of resistance to chloramphenicol, susceptibility to this antimicrobial was determined by disk diffusion for the whole collection. AST results were interpreted following the recommendation of PRAN (AEMPS) and other European authorities (EMA, ESCMID, ECDC), which prioritizes the use of the clinical standard breakpoints established by the European Committee on Antimicrobial Susceptibility Testing (EUCAST 2022) when available. As an alternative, epidemiological cut-off values (ECOFF) or tentative ECOFF (TECOFF) were applied (Table 1). ECOFFs (and TECOFFs) distinguish microorganisms without (wild type) and with phenotypically detectable acquired resistance mechanisms (non-wild type, N-WT) to the agent in question. In addition, the veterinary microbiology laboratory standards from the Clinical & Laboratory Standards Institute (CLSI VET ED5:2020) (CLSI VET01SED5, 2020) and the veterinary recommendations from the Comité de l’antibiogramme de la Société Française de Microbiologie (CASFM VET2020) (CASFM Vétérinaire, 2020) were also visited (Table 1). Based on AST results, the isolates were classified as MDR if displayed resistance to a drug of ≥ 3 of the different antimicrobial categories (Magiorakos et al., 2012).

**Table 1:** Antimicrobials, breakpoints/cut-off values and interpretation reviewed in the present study.

Antimicrobial	^a^Breakpoints CLSI VET ED5:2020			^b^Breakpoints CASFM (VET) 2020		^c^EUCAST 2022 *(T)ECOFF	^d^Breakpoints/cut-off values applied in this study		
	“R”	“I”	“S”	“R”	“S”	“N-WT” >	AST Standards	“R”	“S”
Ampicillin	≥32 (humans)	16	≤8	–	–	8	EUCAST 2022 CLIN	>8	≤8
Amoxicillin-Clavulanic Acid	≥32/16 (humans)	16/8	≤8/4	>16/8	≤4/2	–	EUCAST 2022 CLIN	>8	≤8
Ticarcillin/Clavulanic Acid	–	–	–	–	–	(16)	EUCAST 2022 CLIN	>16	≤8
Cefalexin (1ª)	≥32 (humans) from cefazolin	–	≤16	>32	≤8	(32)	EUCAST 2022 CLIN	>16	≤16
Cefalothin (1ª)	–	–	–	–	–	32	EUCAST 2022 ECOFF	>32	≤32
Cefoperazone (3ª)	–	–	–	>32	≤4	(1)	EUCAST 2022 TECOFF	>1	≤1
Ceftiofur (3ª)	≥ 8 (swine; respiratory; *S*. Choleraesuis)	4	≤2	>4	≤2	1	EUCAST 2022 ECOFF	>1	≤1
Cefquinome (4ª)	-	–	–	>4	≤2	–	CASFM 2020	>4	≤2
Imipenem	≥4 (humans)	2	≤1	–	–	0.5	EUCAST 2022 CLIN	>4	≤2
Flumequine	–	–	–	>8	≤4	2	EUCAST 2022 ECOFF	>2	≤2
Enrofloxacin	–	–	–	>2	≤2	0.125	EUCAST 2022 ECOFF	>0.125	≤0.125
Marbofloxacin	–	–	–	>2	≤2	–	CASFM 2020	>2	<=2
Gentamicin	≥16 (humans)	8	≤4	>4	≤2	2	EUCAST 2022 CLIN	>2	≤2
Neomycin	–	–	–	>16	≤8	8	EUCAST 2022 ECOFF	>8	≤8
Tetracycline	≥16 (humans)	8	≤4	>8	≤4	8	EUCAST 2022 ECOFF	>8	≤8
Polymyxin B / Colistin	–	–	–	>2	≤2	2	EUCAST 2022 CLIN	>2	≤2
Trimethoprim/Sulfamethoxazole	≥4/76 (humans)	–	≤2/38	> 8/152	≤ 2/38	0.5	EUCAST 2022 CLIN	>4	≤2
Florfenicol-MIC (N = 42)	≥ 16 (swine; respiratory; *S*. Choleraesuis)	8	≤4	–	–	16	EUCAST 2022 ECOFF	>16	≤16
Florfenicol-disk 30 μg (N = 42)	–	–	–	<19	≥19	–	CASFM 2020	<19	≥19
Chloramphenicol-disk 30 μg (N = 106)	≤12	13–17	≥18	<19	≥19	–	EUCAST 2022 CLIN	<17	≥17

a “R,” resistant (comments on the species in which it is applied; pathological process; microorganism); “I,” intermediate; “S,” susceptible; −, not available.

b “R,” resistant; “S,” susceptible; −, not available.

c Epidemiological cut-off values (ECOFF) and tentative epidemiological cut-off values *(TECOFF); “N-WT,” non-wild type; −, not available.

d EUCAST 2022 CLIN, results were interpreted, on a priority basis, according to the clinical (CLIN) breakpoints of the European Committee on Antimicrobial Susceptibility Testing (AST) EUCAST 2022; alternatively, ECOFF or TECOFF were applied; and if there was no option, CLSI VET ED5:2020 and CASFM VET2020 breakpoints were visited; “R,” resistant; “S,” susceptible. Numerical values are expressed in mg/l.

### Screening and typing of *mcr* and ESBL genes

By PCR, the presence of *mcr*-1, *mcr*-2, *mcr*-3, *mcr*-4 and *mcr*-5 genes was screened within the collection as described elsewhere (García et al., 2018). Likewise, genetic identification of the ESBLs was performed using the SHV, CTX-M-1 and CTX-M-9 group-specific primers followed by amplicon sequencing (García-Meniño et al., 2018, 2021) (Supplementary Table S4).

### Whole genome sequencing, assembly, and *in silico* analysis

Since the prevalent global ExPEC lineages implicated in human and animal infections, such ST131, belong to phylogroup B2 (Mora et al., 2013; Flament-Simon et al., 2020b), one isolate assigned by PCR to this phylogroup was further investigated by WGS. DNA was extracted and quantified as detailed in García-Meniño et al. (2019). Briefly, DNA was extracted with the DNeasey Blood and Tissue Kit (Qiagen, Hilen, Germany) according to the manufacturer’s instructions. After extraction, the DNA was quantified by an Invitrogen Qubit fluorimeter (Thermo Fisher Scientific, Massachusetts) and assessed for purity using a NanoDrop ND-1000 (Thermo Fisher Scientific, Massachusetts). DNA sequencing was performed using Illumina technology with a NovaSeq 6,000 S4PE150 XP system to obtain 150 bp paired-end reads at Eurofins Genomics (Eurofins Genomics GmbH, Konstanz, Germany), after a standard library preparation (unique dual indexing, that has distinct, unrelated index sequences for each of the i5 and i7 index reads). The quality of the paired-end Illumina reads was evaluated using FastQC. The reconstruction of the genome and *in silico* analysis was performed as described elsewhere (García-Meniño et al., 2019). Briefly, the raw reads were assembled with the VelvetOptimiser.pl. script implemented in the “on line” version of PLAsmid Constellation NETwork (PLACNETw). PLACNETw is a tool implemented for the reconstruction of plasmids from next-generation sequence pair-end. It allows the manual pruning of the graph representation.^1^ The assembled contigs, with genomic size 5.0 Mbp (Supplementary Table S5), were analyzed using the bioinformatics tools of the Center for Genomic Epidemiology (CGE)^2^ as specified, and applying the thresholds suggested by default when required (minimum identity of 95% and coverage of 60%): for the presence of acquired genes and or chromosomal mutations mediating antimicrobial resistance (ResFinder 4.1.), for identification of acquired virulence genes (VirulenceFinder 2.0), plasmid replicon types (PlasmidFinder 2.1/pMLST 2.0), and identification of clonotypes (CHTyper 1.0), and serotypes (SeroTypeFinder 2.0). For the phylogenetic typing, two different MLST (2.0) schemes were applied. Additionally, cgMLSTFinder1.2 was applied for the core genome multi-locus typing (cgMLST) from the raw reads of the isolates. The bacteria’s pathogenicity towards human hosts was predicted using PathogenFinder 1.1.

### Statistical analysis

Pairwise comparisons were performed by a two-tailed Fisher’s exact probability test,^3^ namely: phylogroup distribution was correlated with production stage (lactation *vs* transition), with pathogenic status (commensal *vs* pathogenic group), and MDR. In addition, correlations between pathotypes and colonization factors with respect to the three stages of production, and differences between commensal and pathogenic isolates regarding antimicrobial resistance, *mcr*- and ESBL-production, were also compared. *p* values < 0.05 were considered statistically significant.

## Results

In this study, 93 individual consecutive fecal samples from diarrheal pigs were received between September 2020 and April 2022, which were subjected to the routine diagnosis of colibacillosis at the LREC, USC. As a result, the confluent growth of LMAC plates from 42 individuals, as well as the isolated colonies (up to 10), tested negative by PCR for all virulence traits (toxins, intimin, and fimbriae) associated with diarrheagenic *E. coli* pathotypes. One *E. coli*-like of those negative colonies, classified as commensal *E. coli*, was included here for comparative purposes. On the other hand, 51 individuals (54.8%) tested positive on the confluent growth for any of the virulence traits analyzed here, with the recovery of 64 pathogenic *E. coli* (from 10 samples, up to 3 different isolates were detected based on their distinct virulence-gene profiles) (Table 2; Supplementary Table S6, columns A, B, and S–AK). From the voluntary questionnaire carried out on the use of antibiotic therapy, we learned that 93.2% of farms reported the use of fluoroquinolones (enrofloxacin/marbofloxacin), 63.5% ceftiofur, 44.6% neomycin, 29.7% sulfonamides, 10.8% colistin, 9.5% florfenicol, and 9.5% tiamulin (Supplementary Table S6; columns L–R).

**Table 2:** Origin of samples, and main characteristics of the *Escherichia coli* collection analyzed in this study.

Samples and isolates			Commensal *E. coli*		Pathogenic *E. coli*: No. isolates and main characteristics									Pathotypes	Main colonization factors		Other VF
					Production stage (approx. age range) No. animals sampled		No. isolates	No. isolates	Phylogroups (No.; %)
No. isolates	Phylogroups (No.; %)	ETEC No. (%)	STEC No. (%)	ETEC /STEC No. (%)	aEPEC No. (%)	F4 (K88) No. (%)	F18 No. (%)
Lactation-pre weaning stage (1 day-4 weeks) n = 38		41	25	A (10; 40%) B1 (11; 44%) C (2; 8%) D (1; 4%) E (1; 4%)	16	A (4; 25%) B1 (7; 43.7%) D (2; 12.5%) E (3; 18.7%)	13 (81.2%)	0	0	1 (6.2%)	4 ETEC (25%)	1 ETEC (6.2%)	2 (F41)
Transition-growing stage (>4–9 weeks) n = 49		56	16	A (2; 12.5%) B1 (5; 31.2%) B2 (1; 6.2%) C (6; 37.5%) E (2; 12.5%)	40	A (21; 52.5%) B1 (5; 12.5%) C (1; 2.5%) D (1; 2.5%) E (12; 30%)	32 (80.0%)	4 (10.0%)	3 (7.5%)	1 (2.5%)	11 ETEC (27.5%)	22: 16 ETEC, 3 ETEC/STEC, 3 STEC (55%)	0
Fattening-finishing stage (>9 weeks) n = 6		9	1	A (1; 100%)	8	A (6; 75%) B1 (1; 12.5%) E (1; 12.5%)	5 (62.5%)	1 (12.5%)	0	2 (25%)	1 ETEC (12.5%)	3 ETEC (37.5%)	0
**TOTAL**	**93**	**106**	**42**	**B1 (16; 38.1%)** **A (13; 30.9%)** **C (8; 19%)** **E (3; 7.1%)** **B2 (1; 2.4%)** **D (1; 2.4%)**	**64**	**A (31; 48.4%)** **E (16; 25%)** **B1 (13; 20.3%)** **D (3; 4.7%)** **C (1; 1.6%)**	**50** **(78.1%)**	**5** **(7.8%)**	**3** **(4.7%)**	**4** **(6.2%)**	**16** **(25.0%)**	**26** **(40.6%)**	**2** **(3.1%)**

### Pathogenic *Escherichia coli*: Virulence profiles, phylogroups, and pairwise comparisons

ETEC was the most prevalent pathotype (78.1%) found among the 64 diarrheagenic isolates, positive for enterotoxin-encoding genes (*eltA* and/or *estA*, and/or *estB*). The remaining isolates were assigned to STEC pathotype (7.8%) positive for *stx2e* (Shiga toxin); aEPEC (6.2%) positive for *eae*; and ETEC/STEC (4.7%) positive for both Shiga toxin and enterotoxin-encoding genes. Additionally, two isolates did not fit the definition of any pathotype since tested positive only for F41 fimbriae. Regarding the other intestinal colonization factors, F18 was the most prevalent fimbriae detected (40.6%), followed by F4 (25.0%). Overall, the most common virulence profiles determined (> 5 isolates) were: STa, STb, LT, F18 (23.4%); STa, STb, F4 (14.1%); STb (14.1%) and STb, LT, F4 (9.4%) (Supplementary Table S6, columns T–AF; Table 2). Pairwise comparisons of pathotypes and colonization factors for the three stages of production, only showed a statistically significant association (*p* < 0.05) for F18 fimbria with the fattening and transition isolates.

By the quadruplex PCR described by Clermont et al. (2013, 2019), 5 phylogroups (A, B1, C, D, and E) were identified. Of them, phylogroup A was the most prevalent (48.4%) followed by E (25%) and B1 (20.3%). Prevalence differences were observed by origin of isolation, specifically within the isolates recovered from lactating pigs, where B1 was more prevalent (43.7%), followed by A (25%) (Supplementary Table S6, column AK; Supplementary Table S7; Table 2).

### Commensal *Escherichia coli*: Phylogroups and pairwise comparisons

The group of 42 *E. coli* negative for all virulence-associated genes with porcine diarrheagenic pathotypes exhibited 6 phylogroups (A, B1, B2, C, D, and E). Of them, B1 (38.1%) was the most prevalent, followed by A (30.9%) and C (19%) (Table 2). Pairwise comparisons regarding origin of isolation showed a significant difference (*p* < 0.05) in prevalence of phylogroup C (8% lactation *vs* 37.5% transition).

Overall, the commensal *E. coli* exhibited a similar phylogenetic structure to that of the pathogenic group, but with significant differences in the prevalence of B1, C, and E (38.1 *vs* 20.3%; 19 *vs* 1.6% and 7.1 *vs* 25%, respectively) (Supplementary Table S7). Besides, phylogroup B1 was significantly associated with the lactating group of isolates.

### Antimicrobial susceptibility and genotypic characterization of ESBL and *mcr* genes

MIC values determined for the 106 *E. coli* isolates showed the highest levels of resistance (> 50%) to ampicillin, tetracycline, enrofloxacin (non-wild type, N-WT), flumequine (N-WT), and trimethoprim/sulfamethoxazole (Table 3). Noticeably, we found resistant/N-WT isolates for all antibiotics, except for imipenem (category A of the EMA). Broad-spectrum cephalosporins and polymyxin resistances (category B of the EMA) were exhibited by 16–21.7 and 8% of isolates, respectively. Additionally, disk diffusion assay revealed that 50.9% of the 106 *E. coli* isolates were chloramphenicol resistant. Lastly, the parallel assay for florfenicol (MICs and disk diffusion) performed for a representative group of 42 isolates showed moderate discrepancies (31% *vs* 38.1% of N-WT/resistant isolates, respectively), affecting AST results close to the cut-off/breakpoint values (MIC > 16 N-WT; disk-diffusion *R* < 19, respectively) (Table 3; Supplementary Table S6, columns AL–BY). Comparing the two groups of commensal isolates *vs* pathogenic *E. coli*, only two significant differences were observed, namely, against neomycin (16.7 *vs* 53.1, respectively) and polymyxin B (0 *vs* 12.5%, respectively; *p* < 0.05).

**Table 3:** Antimicrobial resistances and pairwise comparisons.

^a^Antimicrobial	^b^EMA	^c^Breakpoints/cut-off values applied in this study		“R”		Total Analyzed (*N* = 106)		Commensal *E. coli* (*N* = 42)		Pathogenic *E. coli* (*N* = 64)		^d^Two-tailed *p* value
						No “R”	% “R”	No. “R”	%“R”	No. “R”	% “R”	
Ampicillin	D	EUCAST 2022 CLIN	>8	90	**84.9**	36	**85.7**	54	**84.4**	1.00
Amoxicillin–Clav. Acid	C	EUCAST 2022 CLIN	>8	3	2.8	2	4.8	1	1.6	0.56
Ticarcillin/Clav. Acid	C	EUCAST 2022 CLIN	>16	11	10.4	4	9.5	7	10.9	1.00
Cefalexin (1^a^)	C	EUCAST 2022 CLIN	>16	23	21.7	13	31.0	10	15.6	0.09
Cefalothin (1^a^)	C	EUCAST 2022 ECOFF	>32	24	22.6	13	31.0	11	17.2	0.15
Cefoperazone (3^a^)	B	EUCAST 2022 (T)ECOFF	>1	17	16.0	9	21.4	8	12.5	0.28
Ceftiofur (3^a^)	B	EUCAST 2022 ECOFF	>1	23	21.7	13	31.0	10	15.6	0.09
Cefquinome (4^a^)	B	CASFM 2020	>4	17	16.0	9	21.4	8	12.5	0.28
Imipenem	A	EUCAST 2022 CLIN	>4	0	0.0	0	0.0	0	0.0	-
Flumequine	B	EUCAST 2022 ECOFF	>2	78	**73.6**	29	**69.0**	49	**76.6**	0.50
Enrofloxacin	B	EUCAST 2022 ECOFF	>0.125	81	**76.4**	30	**71.4**	51	**79.7**	0.36
Marbofloxacin	B	CASFM 2020	>2	32	30.2	16	38.1	16	25.0	0.19
Gentamicin	C	EUCAST 2022 CLIN	>2	33	31.1	10	23.8	23	35.9	0.21
Neomycin	C	EUCAST 2022 ECOFF	>8	41	38.7	7	16.7	34	53.1	**0.00**
Tetracycline	D	EUCAST 2022 ECOFF	>8	82	**77.4**	31	**73.8**	51	**79.7**	0.64
Polymyxin B	B	EUCAST 2022 CLIN	>2	8	7.5	0	0.0	8	12.5	**0.02**
Trimethoprim/Sulfamethoxazole	D	EUCAST 2022 CLIN	>4	69	**65.1**	24	**57.1**	45	**70.3**	0.21
Florfenicol-MIC (*N* = 42)	C	EUCAST 2022 ECOFF	>16	13	31.0	4	21.1	9	39.1	0.32
Florfenicol-disk 30 μg (*N* = 42)	C	CASFM 2020	<19	16	38.1	7	36.8	9	39.1	1.00
Chloramphenicol-disk 30 μg (*N* = 106)	C	EUCAST 2022 CLIN	<17	54	**50.9**	18	42.9	36	**56.3**	0.23

a Minimal inhibitory concentrations (MICs) of the 106 *E. coli* isolates were determined for all antibiotics showed in the column with the exception of florfenicol, which was tested in parallel by means of MIC and diffusion disk assays for a representative group of 42 isolates. In addition, susceptibility to chloramphenicol was determined in the whole collection by disk diffusion.

b Categorization of antimicrobials proposed by the European Medicines Agency (EMA).

c “R,” resistant breakpoint (EUCAST 2022 CLIN, CLSI VET ED5:2020, CASFM VET2020), or epidemiological cut-off value ((T)ECOFF); highlighted in bold, prevalences ≥ 50%.

d Two-tailed Fisher’s exact probability test; *p* values <0.05 were considered statistically significant (in bold).

Of the 106 isolates, 83 (78.3%) showed resistance to at least one agent in ≥ 3 antimicrobial categories, which were defined as MDR (Magiorakos et al., 2012), with no significant difference between commensal *vs* pathogenic isolates. However, MDR to ≥ 6 antimicrobial categories, were significantly associated with pathogenic *E. coli* (48.4%) *vs* commensal (28.6%) isolates (*p* < 0.05) (Table 4). Regarding phylogroup distribution between the groups of MDR (≥ 3 antimicrobial categories) *vs* non-MDR, the phylogroup C appeared significantly associated with non-MDR isolates (4.8% *vs* 21.7%, respectively; *p* < 0.05) (Supplementary Table S7).

**Table 4:** Prevalence of MDR, ESBL and *mcr* within the 106 *E. coli* and pairwise comparisons.

Status	Total *E. coli* (*N* = 106)		Commensal *E. coli* (*n* = 42)		Pathogenic *E. coli* (*n* = 64)		^a^*p*
	No.	%	No.	%	No.	%	
MDR ≥ 3	83	78.3	32	76.2	51	79.7	0.81
MDR ≥ 6	43	40.6	12	28.6	31	**48.4**	**0.04**
ESBL	23	21.7	13	31.0	10	15.6	0.09
*mcr*	12	11.3	0	0.0	12	**18.8**	**0.00**

a Two-tailed Fisher’s exact probability test. *p* values < 0.05 were considered statistically significant (in bold).

Carriage of ESBL genes was determined in 23 isolates, 13 commensal (31%) and 10 pathogenic (15.6%) *E. coli* (*p* > 0.05), originating from the three stages of production (14 lactation, 8 transition, and 1 from fattening). These 23 isolates were phenotypically predicted as ESBL by the VITEK^®^ 2 system, with resistant MICs for all non-broad spectrum cephalosporins (23 isolates) and for the broad-spectrum cephalosporins: ceftiofur (23 isolates), cefoperazone (17 isolates), and cefquinome (17 isolates). The ESBL-typing by sequencing revealed the presence of SHV and CTX-M genes in 6 and 17 isolates, respectively. Most of the CTX-M positive sequences were classified as group 1 (CTX-M-1, 2 isolates; CTX-M-15, 2 isolates; CTX-M-32, 7 isolates; CTX-M-55, 1 isolate; one could not be typed), and 4 belonged to CTX-M of group 9 (CTX-M-14) (Table 4; Supplementary Table S6, columns CB–CG).

The 106 isolates were also screened for the presence of *mcr-1* to 5 genes, which determined that 12 isolates (11.3%) were *mcr* carriers (8 *mcr*-1 and 4 *mcr*-4), all conforming the ETEC pathotype. These *mcr*-positive isolates were recovered from the lactation and transition subgroups (3 and 9 isolates, respectively). Interestingly, 2 *mcr*-1 and 1 *mcr*-4 isolates were also ESBL-carriers (CTX-M-32 and CTX-M14, respectively). Phenotypically, 8 out of the 12 *mcr*-positive isolates exhibited resistance to polymyxins (MICs > 2), and all 32 isolates, positive for ESBL and/or *mcr*, exhibited MDR (Table 4; Supplementary Table S6, columns AL–CG). Notably, 10 ESBL isolates (2 commensal and 8 pathogenic *E. coli*) displayed MDR to ≥ 8 categories, which were recovered from the three stages of production (3 lactation, 6 transition and 1 fattening) (Supplementary Table S6, columns BZ–CG).

Supplementary Table S8 compares AST results obtained by applying breakpoints/cut-off values from different standards (EUCAST 2022, TECOFF 2022, CLSI VET ED5:2020, and CASFM VET2020). Clinical breakpoints are missing within the three standards for veterinary antibiotics such as cefquinome, marbofloxacin, or florfenicol.

### *In silico* characterization: O4:H5-B2-ST12 (CH13-223)

The PCR screening of *rfb*O25, performed to presumptively detect the pandemic ST131 clonal group of phylogroup B2, gave no positive result. However, this phylogroup was determined in one commensal isolate, which was further investigated by WGS to determine its virulence and potential zoonotic profile. Table 5 shows the analysis of the assembled contigs using the bioinformatics tools of the CGE (see footnote 2). Briefly, SeroTypeFinder predicted O4:H5 antigens, MLST tools assigned the sequence type (ST)12 of the Achtman 7-gene scheme and core genome ST (cgST) 44799 based on the Enterobase database, and CHTyper predicted the clonotype (CH)13–223, so that the clonal group eventually assigned to this isolate was O4:H5-B2-ST12 (CH13-223). The resistome analysis revealed the absence of chromosomal mutations but the presence of encoded mechanisms of acquired antibiotic resistance for beta-lactams (*bla*_TEM_), aminoglycosides (*aph(3″)-Ib, aph(6)-Id*), sulphonamides (*sul2*) and tetracycline *tet*(A), which correlated with the phenotypic expression of resistance against ampicillin and tetracycline. VirulenceFinder revealed virulence traits associated with ExPEC lineages. In fact, the virulence profile predicted for this isolate conforms the status of extraintestinal pathogenic *E. coli* (ExPEC status ≥ 2 of these five virulence traits: *papA* and/or *papC*, *afa/dra*, *sfa/foc*, *iutA*, *kpsM* II) (Johnson et al., 2003), and the status of uropathogenic *E. coli* (UPEC status ≥3 of these four traits: *chuA*, *fyuA*, *vat* and *yfcV*) (Spurbeck et al., 2012). Furthermore, this genome was predicted as human pathogen (probability 88%) when analyzed with PathogenFinder. Regarding plasmid replicon types, PlasmidFinder identified an IncQ1 with 100% identity but with a coverage of 529/796. The PLACNET genome reconstruction (Supplementary Figure S1) showed a MOBQ relaxase within a 977 kb chromosomal contig, implying the presence of a potential integrative and conjugative element (ICE). Besides, a plasmid replication initiator protein (RIP) was identified in a node of 4.6 kb linked to plasmid and chromosomal references.

**Table 5:** *In silico* characterization of the B2 commensal *E. coli.*

^a^ID code for isolate/genome	^b^O:H antigens	^c^ST#1/ST#2	^d^cgST	^e^CHType	^f^Acquired resistances	^g^Plasmid replicon	^h^Virulence genes	^i^Mobile genetic elements (and relation to AMR and virulence traits)	^j^Predicted as human pathogen (probability)
FVL196 /LREC_294	O4:H5	12 / 36	44799	13–223	*bla*_TEM-1B_, *aph(3″)-Ib*, *aph(6)-Id*, *sul2, tet(A)*, *sitABCD*	IncQ1	*cea, chuA, clbB, cnf1, focC, focC/sfaE, fyuA, gad, hra, iroN, irp2, iss, kpsE, kpsMII, mchB, mchC, mchF, mcmA, ompT, papA_F11, papA_F14, papC, sfaD, sitA, tcpC, terC, usp, vat, yfcV*	IncQ1 (*aph (3″)-Ib, aph(6)-Id, sul2*), ISEc41 (*kpsE*, *terC*, *kpsMII*), ISEc40-ISEc13 (*mchB*, *mchF*, *sfaD*, *focC*, *mchC*, *cea*, *mcmA*, *iroN*), IS682-ISKpn37 (*cnf1*), MITEEc1 (*terC*), ISSen4 (*yfcV*),	Yes (0.88)

a Isolate and genome (LREC) identification.

b O and H antigen prediction with SerotypeFinder 2.0.

c Sequence types (ST#1 and ST#2) based on two different MLST schemes *E. coli* #1 and *E. coli* #2, respectively, and retrieved with MLST 2.0.4.

d Core genome ST obtained with cgMLSTFinder1.1. Software run against the Enterobase database.

e-j Clonotype, acquired antimicrobial resistance genes, plasmid replicon, virulence genes, mobile genetic elements associated with antimicrobial resistance, virulence traits, and the prediction of a bacteria’s pathogenicity towards human hosts were also analyzed by using CHtyper 1.0, ResFinder 4.1, PlasmidFinder 2.1, VirulenceFinder 2.0, MobileElementFinder 1.03, PathogenFinder 1.1 online tools at the Center of Genomic Epidemiology (http://www.genomicepidemiology.org/services/), respectively.

f Resistome: Acquired resistance genes: beta-lactam: *bla*_TEM-1B_, aminoglycosides: *aph(3″)-Ib, aph(6)-Id*; sulphonamides: *sul2*; tetracycline: *tet(A)*; peroxide: *sitABCD* (mediates transport of iron and manganese and resistance to hydrogen peroxide).

g Virulence determinants: *cea:* colicin E1; *chuA*: outer membrane hemin receptor; *clbB*: hybrid non-ribosomal peptide / polyketide megasynthase; *cnf1*: cytotoxic necrotizing factor; *focC*: S fimbrial/F1C minor subunit; *focC/sfaE*: S fimbrial/F1C minor subunit; *fyuA*: siderophore receptor; *gad*: glutamate decarboxylase; *hra*: heat-resistant agglutinin; *iroN*: enterobactin siderophore receptor protein; *irp2*: high molecular weight protein 2 non-ribosomal peptide synthetase; *iss*: increased serum survival; *kpsE*: capsule polysaccharide export inner-membrane protein; *kpsMII:* polysialic acid transport protein group 2 capsule; *mchB*: microcin H47 part of colicin H; *mchC*: MchC protein; *mchF*: ABC transporter protein MchF; *mcmA*: Microcin M part of colicin H; *ompT*: outer membrane protease (protein protease 7); *papA_F11*: major pilin subunit F11; *papA_F14*: major pilin subunit F14; *papC*: outer membrane usher P fimbriae; *sfaD*: S fimbrial/F1C minor subunit; *sitA*: iron transport protein; *tcpC*: Tir domain-containing protein; *terC*: tellurium ion resistance protein; *usp*: uropathogenic specific protein; *vat*: vacuolating autotransporter toxin, *yfcV*: fimbrial protein.

## Discussion

Antibiotic resistance is of great public health concern since the antibiotic-resistant bacteria associated with the animals may be easily transmitted to humans via food chains, and widely disseminated in the environment via animal wastes (sludge-fertilized soils and manure). Intensive pig farming is one of the livestock activities widely recognized for its higher antimicrobial consumption (European Medicines Agency, 2021). The high prevalence of *E. coli* in swine enteric disease, their role as commensal, and their potential spread throughout animal-derived foods, makes this species key for the monitoring of AMR in industrial swine farming. In fact, *E. coli* is the main causative agent implicated in neonatal and PWD, and once the clinical signs appear, antimicrobials are the control solution for colibacillosis (Luppi, 2017). On the other hand, *E. coli* is commonly used as an indicator bacteria on the “One-Health” surveillance of antimicrobial resistance (AMR) by the European Food Safety Authority (EFSA) and European Centre for Disease Prevention and Control (ECDC) (EFSA (European Food Safety Authority) and ECDC (European Centre for Disease Prevention and Control), 2019). On top of that, there is a global consensus on the need for efficient antimicrobial stewardship programs in pigs to reduce the selection of resistant bacteria (Verliat et al., 2021; Bosman et al., 2022; Vilaró et al., 2022). Here, we aimed to gain knowledge on the diagnosis and clinical difficulties in the veterinary sector when providing the best antibiotic therapy option. Another objective here was to obtain up-to-date information on antimicrobial resistance, and *E. coli* pathotypes in Spanish swine production. The protocol applied in the present study has been performed within the diagnosis service of the LREC, covering the same geographical areas of Spain for more than 20 years. Thus, the results obtained here could be compared with our previous data (García-Meniño et al., 2021).

### Antimicrobial susceptibility testing interpretation: challenges without clinical breakpoints

The goal of *in vitro* AST is to inform clinicians whether an antimicrobial is appropriate for the infection caused by a specific isolate. Furthermore, there is no successful option of empiric treatment for *E. coli* due to their heterogeneous MIC profile, so optimization to minimize resistance selection should be based on AST for each clinical case (Cortés et al., 2010; García-Meniño et al., 2019, 2021; Vilaró et al., 2022). However, we report here the lack of standardized breakpoints for many antimicrobials of common use in livestock. Currently, in the EUCAST reference method, there are no clinical breakpoints for cefalothin, cefoperazone, ceftiofur, flumequine, enrofloxacin, neomycin, tetracycline, and florfenicol. For these, we reviewed ECOFF (and TECOFF) values. Since cefquinome, marbofloxacin had neither clinical breakpoints nor cut-off values in EUCAST (nor in CLSI), we took them from CASFM VET2020, which were also applied for florfenicol-disk assay interpretation (Table 1). It is of note that the last version of CLSI and CASFM veterinary standards was reviewed in 2020. The comparison of results using the different standards (Supplementary Table S8), showed a moderate to high correlation between clinical breakpoints and cut-off values, except for enrofloxacin, neomycin, and florfenicol. The discrepancies observed for enrofloxacin and neomycin are explained by lower ECOFF values compared to CASFM VET2020 (> 0.125 *vs* > 2 and > 8 *vs* > 16, respectively). This observation is consistent with the fact that ECOFF values define a microorganism as non-wild type (N-WT) for a species by the phenotypical detection of an acquired or mutational resistance mechanism to the drug in question, while clinical breakpoints define a microorganism as resistant by a level of antimicrobial activity associated with a high likelihood of therapeutic failure. Nevertheless, it is outstanding the correlation obtained for flumequine applying CASFM VET and ECOFF (64.2% resistant and 76.4% N-WT, respectively), in comparison with data on enrofloxacin (31.1% resistant and 76.4% N-WT, respectively). Besides, resistance prevalence to marbofloxacin is expected to correlate with enrofloxacin (Vilaró et al., 2022). In our previous study (2005–2017), where the CLSI2020 clinical breakpoints were applied, a high percentage of isolates implicated in colibacillosis exhibited quinolone/fluoroquinolone resistance: 82.3% to nalidixic acid, 56.5% to ciprofloxacin and 48.4% to levofloxacin. To note, 93.2% of the farms involved in the present study reported the use of fluoroquinolones, and 44.6% reported the use of neomycin. Although applying ECOFF values would greatly restrict the therapeutic possibilities of neomycin and enrofloxacin, the high percentage of N-WT determined in both (> 70% commensal and pathogenic *E. coli* for enrofloxacin; 16.7% commensal and 53.2% of pathogenic *E. coli* for neomycin) would indicate an overuse, which might compromise therapeutic use soon. The ATS analysis revealed another critical point, which is the fixed range of drug concentrations provided by commercial kits for MIC determination. This greatly limits the flexibility of standard applications, as shown in Supplementary Table S8 for cefoperazone, and trimethoprim/sulfamethoxazole.

Regarding phenicols, we conducted a comparative florfenicol AST using MIC and disk diffusion in 42 isolates. In addition, susceptibility to chloramphenicol was determined in the entire collection by disk diffusion. By applying the clinical EUCAST breakpoint for chloramphenicol, we observed a similar high resistance prevalence (around 50%) compared to the previous study, with no discrepancies within standards. However, contradictory differences were observed in florfenicol resistance (50% “R” *vs* 31% N-WT, according to CLSI VET 2020 and ECOFF values, respectively). Phenicols are broad-spectrum antimicrobials used in veterinary medicine, although chloramphenicol was banned in EU in 1994 for its use in food-producing animals due to its toxicity and side effects for humans. The resistance level to chloramphenicol maintained over time (observed in this and previous study) can be probably due to different molecular mechanisms conferring resistance to both non-fluorinated (e.g., chloramphenicol) and fluorinated (e.g., florfenicol). Besides, genes encoding phenicol resistance (*cat*A1, *cml*A, and *flo*R) are often carried by plasmids together with other resistance genes. Co-selection under the selective pressure of non-phenicol antimicrobial drugs has been reported (Poirel et al., 2018). Florfenicol in pigs is indicated for the treatment of swine respiratory disease caused by *Actinobacillus pleuropneumoniae* and *Pasteurella multocida*. The CLSI 2020 references the breakpoint for *S. enterica* subsp. *enterica* serovar Choleraesuis “R” > =16, and the suggested ECOFF value for *E. coli* to detect an N-WT is > 16.

The high prevalence of MDR to ≥ 3 antimicrobial categories (> 75%) observed in our study is of concern. Furthermore, no differences were observed between the groups of commensal and pathogenic isolates, which would indicate that the therapeutic administration is exerting selective and permanent pressure on the commensal population (Zeineldin et al., 2019). On the other hand, the finding of MDR to ≥ 6 categories associated with pathogenic isolates, still implies a greater selective pressure on commensals which might incorporate resistance genes or could be displaced by resistant bacteria with the successful stabilization in the porcine microbiota. Strict control measures are necessary to avoid transmission (MDR bacteria or genes) to humans via food, or to the environment through animal wastes.

### Phylogeny, *mcr* and ESBL genes: Monitoring tool

Since the *E. coli* phylogroups are not randomly distributed, the phylogroup assignment described by Clermont et al. (2013); Clermont et al. (2019) is a simple and valuable method to analyze population changes, and even for clinical purposes. Different factors define the phylogeny, such as environment, physiology, diet, and maturation of the digestive tract, host status, or virulence-gene carriage of the bacteria (Escobar-Paramo et al., 2006; Cortés et al., 2010; Clermont et al., 2013). Thus, we apply this method in the diagnostic routine to monitor the evolution of pathogenic clones. The structure of *E. coli* in pigs is quite homogeneous worldwide, and within commensal pig *E. coli*, the predominance of phylogroup A, followed by B1 has been reported by different authors (Bok et al., 2013; Reid et al., 2017). Here, we found a similar phylogenetic population structure both in commensal and pathogenic isolates, which were assigned to A, B1, C, D, and E (additionally, one commensal isolate showed phylogroup B2). However, significant differences were detected in the prevalence of certain phylogroups associated with commensal (B1 and C) or pathogenic (E). Bok et al. (2020) reported wider diversity, including in addition the detection of F, or B2. The B2 phylogroup was determined by Bok et al. (2020) in 4.5% of piglets and 2.4% of sows. B2 is a phylogroup rarely reported in domestic pigs, which in our previous surveys was found in 3.6% of 499 pig isolates, associated with the pandemic clonal group ST131. In the present study, we did not recover any B2-ST131 isolate but the phylogroup B2 was determined in one commensal *E. coli*. The *in silico* characterization revealed the presence of clone O4:H5-B2-ST12 (CH13-223). The virulome analysis showed the carriage of various traits associated with extraintestinal virulence and, notably, its virulence profile conforms both, the ExPEC and UPEC status. Besides, it was predicted as human pathogen. ST12 is one of the global ExPEC lineages according to Manges et al. (2019), which is frequently implicated in extraintestinal disease, in both humans and domestic animals (mainly dogs, cats, and pigs) (Spindola et al., 2018; Flament-Simon et al., 2020b; Kidsley et al., 2020; Carvalho et al., 2021). The finding of this B2 clone, likewise our previously reported ST131 *mcr*-1 isolates (García-Meniño et al., 2018), deserves special attention. It would be indicative of swine as a potential reservoir of ExPEC not only for livestock but for humans too (Zhu et al., 2017; Bok et al., 2020; Flament-Simon et al., 2020b). Extraintestinal infections by *E. coli* such as urinary tract infections (UTI) also cause severe losses in the swine industry, mainly linked to antibiotic therapy, early disposal of breeding sows, or acute death when severely affected (Spindola et al., 2018). Therefore, specific clones such as O4:H5-B2-ST12 (CH13-223), or O25b:H4-B2-ST131 (CH40-374/161) of virotype D5 (García-Meniño et al., 2018; Flament-Simon et al., 2020a), should be monitored under a “One-Health” perspective.

Regarding the present collection of 64 pathogenic isolates, we found here a higher phylogenetic diversity, with a shift in prevalences compared to previous data. While the prior study (2005–2017) on pathogenic *E. coli* clearly showed the predominance of phylogroup A (85.5%), followed by E (12.4%) and B1 (2.1%), the present collection (2020–2022) exhibited a different distribution: phylogroup A (48.4%), followed by E (25%), B1 (20.3%), D (4.7%) and C (1.6%). These changes in prevalence, and therefore, in the predominant clones involved in swine colibacillosis, were already observed by us in Spain between the periods 2005–2017 and 1986–1991 (García-Meniño et al., 2021). This fact might be explained by the selective pressure derived from different antimicrobial therapies and vaccination programs applied over time.

An outstanding finding in the present study, is the important decrease in colistin-resistant *mcr*-bearing isolates, from 76.9% within 186 PWD isolates of the period 2005–2017 to 11.3% (present study, within 106 *E. coli*; *p* < 0.05). Besides, we have found *mcr* genes exclusively associated with pathogenic isolates (11.3% *vs* 0% of commensals; *p* value < 0.05), and the result is still significant if we compare data only from the group of diarrheagenic isolates: 12 isolates from 64 *E. coli*, 18.7% (2020–2022) *vs* 143 out of 186, 76.9% (2005–2017). Differently to our first study, where we observed co-occurrence of *mcr*-1/*mcr*-4, *mcr*-1/*mcr*-5, and *mcr*-4/*mcr*-5 in a significant number of pathogenic pig isolates (García et al., 2018; García-Meniño et al., 2019), the 12 *mcr*-positive isolates recovered in the period 2020–2022, show single *mcr* carriage (*mcr*-1 or *mcr*-4). Ours would be a unique study, analyzing not only *mcr*-1 but also other prevalent *mcr* genes in swine, in both commensal and pathogenic *E. coli*. The mandatory monitoring in the EU under Commission Implementing Decision (EU) 2020/1729 [Commission Implementing Decision (EU), 2020] is based on phenotypic susceptibility only, and it does not discriminate between different colistin resistance mechanisms. From our point of view, molecular testing is required to confirm the underlying mechanisms of resistance and to gain understanding on the epidemiology of *mcr*-positive *E. coli*. Besides, if we apply the standardized phenotypic assays and current clinical cut-off (> 2 mg/l), many *mcr*-positive *E. coli* (especially those of the *mcr*-1 type) remain undetected (García-Meniño et al., 2020). This would be the case of 4 out of the 12 *mcr*-1 *E. coli* in the present study. Another limitation of the Decision (EU) 2020/1729 [Commission Implementing Decision (EU), 2020], is that surveys only refer to indicator commensal *E. coli*. We suggest the monitoring of pathogenic (invasive) *E. coli* in surveillance programs to accurately determine the persistence of colistin resistance. Our findings on the decrease in colistin-resistant *mcr*-bearing isolates would reinforce the conclusions of longitudinal studies on the *mcr*-1 abundance in food-producing pigs, and its relationship with the use of polymyxins (Wang et al., 2020; Miguela-Villoldo et al., 2022). Thus, Miguela-Villoldo et al. (2022) reported the decreasing trend of colistin resistance associated with *mcr*-1 gene in Spain, since the EMA and AEMPS strategies were applied in 2016 to reduce colistin use in animals. Likewise, Wang et al., 2020 showed a substantial reduction in sales of colistin in animals (2015–2018), after the withdrawal of colistin as growth promoter in China in 2017, which was rapidly followed by a significant reduction in the prevalence of *mcr*-1 in both the animal and human sectors. Despite the decrease of antibiotic selective pressure due to the colistin consumption reduction, the effect of co-selection by other antimicrobials, such as broad-spectrum cephalosporins, could be causing the persistence of colistin resistance, as recognized in the joint report of the ECDC, EMA and the European Food Safety Authority (EFSA; JIACRA III) (European Centre for Disease Prevention and Control (ECDC); European Food Safety Authority (EFSA); European Medicines Agency (EMA), 2021). In the present study, 23 of the 106 isolates (21.7%) showed resistance to ceftiofur, and the 23 were positive for ESBL genes, without significant difference in commensal *vs* pathogenic groups (31% *vs* 15.6%; *p* > 0.05). The AST analysis of our previous study (2005–2017) determined that 17 (9%) isolates were phenotypically identified as ESBL-producers (including 11 *mcr*-positive isolates), which would mean a significantly lower figure to present data (12 of 64, 18.7% of diarrheagenic *E. coli*; p < 0.05) (García-Meniño et al., 2021). The resistant prevalences showed here, both for commensal and pathogenic strains, might be highlighting an increasing use of cephalosporins, in agreement with the voluntary survey, in which 47 (63.5%) out of 74 farms reported the use of ceftiofur.

## Conclusion

There is an urgent need of harmonized guidelines, including standardized clinical breakpoints for all antimicrobials of common use in veterinary medicine. Our findings show a significant decrease in *mcr* isolates of porcine origin which would correlate with the drastic reduction in the use of colistin in Spain. We suggest that a diagnosis workflow based on AST, detection of *mcr* and ESBL genes, and phylogenetic characterization, would be useful as a monitoring tool for MDR and *E. coli* population shifts, under a “One-Health” perspective.

## Data availability statement

The datasets presented in this study can be found in online repositories. The names of the repository/repositories and accession number(s) can be found in the article/Supplementary material. In addition, the nucleotide sequence of the B2-ST12 LREC_294 genome (FVL196/22 isolate) was deposited in the European Nucleotide Archive (ENA) with the following accession (ERR10033261) and BioSample (SAMEA110466098) codes, as part of BioProject ID PRJEB55192.

## Author contributions

AMo, VGo, AMe, AA, and ER: conceptualization. VGa and IG-M: methodology. VGo, MJ-O, AMo, and AA: software. AMo, VGa, IG-M, VGo, and AMe: formal analysis. AMo, VGa and IG-M: investigation. VGa, IG-M, VGo, MJ-O, AMe, AA, ER and AMo: writing—review and editing. AMo: supervision, project administration, and writing—original draft preparation. All authors contributed to the article and approved the submitted version.

## Funding

This work was supported by grants from the Agencia Estatal de Investigación (AEI, Spain), co-funded by the European Regional Development Fund of the European Union, a Way to Make Europe (ERDF) [project ID. PID2019-104439RB-C21/AEI/10.13039/501100011033] and from the Consellería de Cultura, Educación e Ordenación Universitaria (Xunta de Galicia) and ERDF [grant ID. ED431C 2021/11]. VGa and IG-M acknowledge the Consellería de Cultura, Educación e Ordenación Universitaria, Xunta de Galicia for their post-doctoral grants (Grant Number ED481D-2022-012 and ED481B-2021-006, respectively).

## Conflict of interest

AA and ER were employed by company Laboratorios Syva S.A.

The remaining authors declare that the research was conducted in the absence of any commercial or financial relationships that could be construed as a potential conflict of interest.

## Publisher’s note

All claims expressed in this article are solely those of the authors and do not necessarily represent those of their affiliated organizations, or those of the publisher, the editors and the reviewers. Any product that may be evaluated in this article, or claim that may be made by its manufacturer, is not guaranteed or endorsed by the publisher.

## Footnotes

^1^ https://github.com/LuisVielva/PLACNETw

^2^ https://www.genomicepidemiology.org/services/

^3^ http://vassarstats.net/tab2x2.html
