# Supplementary material for: Mobile colistin resistance (MCR), extended-spectrum beta-lactamase (ESBL) and multidrug resistance monitoring in Escherichia coli (commensal and pathogenic) in pig farming: need of harmonized guidelines and clinical breakpoints
Source: Front Microbiol. 2022 Dec 2;13:1042612. doi: 10.3389/fmicb.2022.1042612 (PMC9756432; doi:10.3389/fmicb.2022.1042612)
Supplement: Supplementary file 1 [file Data_Sheet_1.pdf]

Supplementary Material

Mobile colistin resistance (MCR), extended-spectrum beta-lactamase (ESBL) and multidrug resistance monitoring in *Escherichia coli* (commensal and pathogenic) in pig farming: Need of harmonized guidelines and clinical breakpoints

Vanesa García, Isidro García-Meniño, Verónica Gómez, Miguel Jiménez-Orellana, Antonio Méndez, Alvaro Aguarón, Elisabet Roca, Azucena Mora

Correspondence: azucena.mora@usc.es

PCR reactions

E. coli DNA was extracted from the confluent growth from the fecal swabs plated on lactose MacConkey agar (LMAC, Oxoid) incubated at 37 °C for 18–24 h or from a single overnight-grown colony using the boiling lysis method. Briefly, overnight-grown was picked with a 1 µl inoculation loop and suspended in 600 µl of sterile Milli-Q water. Bacterial suspensions were boiled at 100 °C for 5 min and then centrifuged for 2 min at 11,000 rpm to pellet bacterial debris. The supernatant was used as DNA template in PCR.

All PCR reactions were done in a final reaction volume of 25 µl, using 5 µl of DNA template, 12.5 µl of NZYTaq 2x Green MasterMix, 0.2-0.5 µM of each primer, and up to 25 µl of sterile Milli-Q water. PCR amplifications were performed on Applied Biosystem 2720 Thermal Cycler using an initial heat activation step of 3 min at 94 °C; then 35 cycles of 1 min at 94 °C, 1 min at specific annealing temperature (depending on the primer set used), and 90 min at 72 °C; and an final extension step of 3 min at 72 °C.

PCR products were separated through 1.5% agarose gel (Seakem LE agarose, Lonza) containing Green Safe Premium (Nzytech) (3 µl/100 ml agarose) by convectional electrophoresis and the amplified PCR products were visualized using Gel Doc XR (BioRad, CA).

The primers, the amplicon size and the specific annealing T^a for each PCR reaction are indicated in the Supplementary Tables 1, 2, 3, and 4.

Table S1. Targets and primers associated with diarreagenic pathotypes of *E. coli*

Pathotype	Target	Primers	Nucleotide sequence (5´- 3´)	Size (bp)	Annealing Tª (°C)	Reference
STEC	*stx2e*	Stx2e-F1	CGGAGTATCGGGGAGAGGC	411	58	Scheutz *et al.*, 2012
		Stx2e-R2	CTTCCTGACACCTTCACAGTAAAGGT			
EPEC	*eae*	EAE-V3F	CATTGATCAGGATTTTTCTGGT	510	54	Mora *et al.*, 2011
		EAE-MBR	TCCAGAATAATATTGTTATTACG			
ETEC	*eltA*	LT-A-1	GGCGACAGATTATACCGTGC	696	54-56	Schultsz *et al.*, 1994
		LT-A-2	CCGAATTCTGTTATATATGTC			
ETEC	*estA*	STa-A	ATTTTTATTTCTGTATTGTCTTT	176	50-52	Penteado *et al.*, 2002
		STa-B	GGATTACAACACAGTTCACAGCAGT			
ETEC	*estB*	STb-1	ATCGCATTTCTTCTTGCATC	172	54	Blanco *et al.*, 1997
		STb-2	GGGCGCCAAAGCATGCTCC			
ETEC	F18	F18-F	GTGAAAAGACTAGTGTTTATTC	510	54-56	Imberechts *et al.*, 1992
		F18-R	CTTGTAAGTAACCGCGTAAGC			
ETEC	F4 (K88)	K88-F	GGTGATTTCAATGGTTCGGTC	764	66	Franklin *et al.*, 1996
		K88-R	ATTGCTACGTTCAGCGGAGCG			
ETEC	F5 (K99)	K99-A	CCAGCGCCCGGCAGTAATGACTGC	278	64	Blanco *et al.*, 2006
		K99-B	CCACCATTAGACGGAGCGCGG			
ETEC	F41	F41-A	GGCTATGGAAGACTGGAGAGGG	545	60	Blanco *et al.*, 2006
		F41-RN	GACTGAGGTCATCCCAATTGTGG			García-Meniño *et al.*, 2018
ETEC	F6 (P987)	P987-F	GCGCCCGCTGAAAACAACACCAGC	467	64	Blanco *et al.*, 2006
		P987-R	GTACCGGCCGTAACTCCACCG			

Table S2. Primers used for the detection of *rbf*O25 and *uidA* genes

Target	Primers	Nucleotide sequence (5´- 3´)	Size (bp)	Annealing Tª (°C)	Reference
*rfb*O25b	rfbO25b.r	TGCTATTCATTATGCGCAGC	300	56	Clermont *et al.*, 2008
	rfb.1bis	ATACCGACGACGCCGATCTG			
uidA	uidA-F	GCGTCTGTTGACTGGCAGGTGGTGG	503	60	Gómez-Duarte *et al.,* 2010
	uidA-R	GTTGCCCGCTTCGAAACCAATGCCT			

Table S3. Targets and primers to determine phylogroups

Target	Primers	Nucleotide sequence (5´- 3´)	Size (bp)	Annealing Tª (°C)	Reference
chuA	chuA.1b	ATGGTACCGGACGAACCAAC	288	58	Clermont *et al*., 2013
	chuA.2	TGCCGCCAGTACCAAAGACA			Clermont *et al*., 2000
yjaA	yjaA.1b	CAAACGTGAAGTGTCAGGAG	211		Clermont *et al*., 2013
	yjaA.2b	AATGCGTTCCTCAACCTGTG			
TspE4C2	TspE4C2.1b	CACTATTCGTAAGGTCATCC	152		Clermont *et al*., 2013
	TspE4C2.2b	AGTTTATCGCTGCGGGTCGC			
arpA	AceK.f	AACGCTATTCGCCAGCTTGC	400		Clermont *et al*., 2013
	ArpA1.r	TCTCCCCATACCGTACGCTA			
trpAgpC (C)	trpAgpC.1	AGTTTTATGCCCAGTGCGAG	219	56	Lescat *et al*., 2013
	trpAgpC.2	TCTGCGCCGGTCACGCCC			
arpA (E)	ArpAgpE.f	GATTCCATCTTGTCAAAATATGCC	301	57	Lescat *et al*., 2013
	ArpAgpE.r	GAAAAGAAAAAGAATTCCCAAGAG			
trpA	trpBA.f	CGGCGATAAAGACATCTTCAC	489	56	Clermont *et al*., 2008
	trpBA.r	GCAACGCGGCCTGGCGGAAG			
ybgD (G)	*ybgD.1*	TATGCGGCTGATGAAGGATC	177	59	Clermont *et al*., 2019
	ybgD.2	GTTGACTAAGCGCAGGTCGA			
cfaB (F)	*cfaB.1*	CTAACGTTGATGCTGCTCTG	384		Clermont *et al*., 2019
	cfaB.2	TGCTAACTACGCCACGGTAG			

Table S4. Primers used for the detection and / or sequencing of TEM, SHV, CTX-M and MCR genes

Target	Primers	Nucleotide sequence (5´- 3´)	Size (bp)	Annealing Tª (°C)	Reference
$bla_{\text{CTX-M}}$	CTX-C3	ATGTGCAGCACCAGTAAAGTGATG	542	55	Mora *et al.*, 2013
	CTX-C4	ACCGCGATATCGTTGGTGGTGCC			
$bla_{\text{CTX-M}}$ group1	M13U	GGTTAAAAAATCACTGCGTC	863	60	Saladin *et al.*, 2002
	M13L	TTGGTGACGATTTTAGCCGC			
blaCTX-M-grupo 1	[a]CTX-15-F1	GAAGCTAATAAAAAACACACGTGG	1044-1123	52	Mora *et al.*, 2013
	[a]CTX-15-R	GTATGCGCAAGCGCAGGTGG			
$bla_{\text{CTX-M}}$ group9	CTX-M9-F	GTGACAAAGAGAGTGCAACGG	856	64	Simarro *et al.*, 2000
	CTX-M9-R	ATGATTCTCGCCGCTGAAGCC			
$bla_{\text{CTX-M}}$ group9	[a]CTX-M9-14-14B-24F	GAATACTGATGTAACACGGA	998	44	García-Meniño *et al.*, 2018
	[a]CTX-M9-R	AGCTGAAGATGTATATCAAG			
$bla_{\text{CTX-M}}$ group9	[a]CTX-M9-14-14B-24F	GAATACTGATGTAACACGGA	989	52	García-Meniño *et al.*, 2018
	[a]CTX-M14-24-R	CTGCGTTGTCGGGAAGATACG			
$bla_{\text{CTX-M}}$ group9	[a]CTX-M9-14B-F	CCTATACCCGAGGCGCGACAG	1059	44	García-Meniño *et al.*, 2018
	[a]CTX-M9-R	AGCTGAAGATGTATATCAAG			
$bla_{\text{CTX-M}}$ group9	[a]CTX-M14-24-F	CTAAATTCTTCGTGAAATAGTG	1049	44	García-Meniño *et al.*, 2018
	[a]CTX-M14-24-R	CTGCGTTGTCGGGAAGATACG			
bla_{SHV}	SHV-F2	TTGTCGCTTCTTTACTCGCC	879	64	Mora *et al.*, 2013
	SHV-R2	CCCGGCGATTTGCTGATTTCGC			
mcr-1	mcr1_320bp_fw	AGTCCGTTTGTTCTTGTGGC	320 pb	58	Rebelo *et al.*, 2018
	mcr1_320bp_rev	AGATCCTTGGTCTCGGCTTG			
mcr-2	mcr2_700bp_fw	CAAGTGTGTTGGTCGCAGTT	715 pb		Rebelo *et al.*, 2018
	mcr2_700bp_rev	TCTAGCCCGACAAGCATACC			
mcr-3	mcr3_900bp_fw	AAATAAAAATTGTTCCGCTTATG	929 pb		Rebelo *et al.*, 2018
	mcr3_900bp_rev	AATGGAGATCCCCGTTTTT			
mcr-4	mcr4_1100bp_fw	TCACTTTCATCACTGCGTTG	1116 pb		Rebelo *et al.*, 2018
	mcr4_1100bp_rev	TTGGTCCATGACTACCAATG			
mcr-5	MCR5 FV	ATGCGGTTGTCTGCATTTATC	1644 pb		Borowiak *et al.*, 2017
	MCR5 RV	TCATTGTGGTTGTCCTTTTCTG			

[a]Primers used for sequencing.

Table S5. General parameters of the assembly B2 commensal *E. coli* genome

**Isolate**	LREC_294
**Kmer**	131
**Roadmap file size**	538667191
**Total number of contigs**	81
**N50**	319,990
**Length of longest contig (bp)**	976,860
**Total bp in contigs**	5,082,346
**Number of contigs > 1kb**	38
**Total bases in contigs > 1kb**	5,065,263
**Library**	334 +/- 103
**Coverage**	35x
**ENA accession number**	ERS12564129

References

Blanco, M., Blanco, J.E., Gonzalez, E.A., Mora, A., Jansen, W., Gomes, T.A., Zerbini, L.F., Yano, T., de Castro, A.F., Blanco, J. (1997). Genes coding for enterotoxins and verotoxins in porcine *Escherichia coli* strains belonging to different O:K:H serotypes: relationship with toxic phenotypes. *J. Clin. Microbiol.* 35, 2958-2963. DOI: 10.1128/jcm.35.11.2958-2963.1997.

Blanco, M., Lazo, L., Blanco, J.E., Dahbi, G., Mora, A., López, C., González, E.A., Blanco, J. (2006). Serotypes, virulence genes, and PFGE patterns of enteropathogenic *Escherichia coli* isolated from Cuban pigs with diarrhea. *Int. Microbiol.* 9, 53-60.

Borowiak, M., Fischer, J., Hammerl, J.A., Hendriksen, R.S., Szabo, I., Malorny, B. (2017). Identification of a novel transposon-associated phosphoethanolamine transferase gene, *mcr-5*, conferring colistin resistance in d-tartrate fermenting *Salmonella enterica* subsp. *enterica* serovar Paratyphi B. *J. Antimicrob. Chemother.* 72, 3317-3324. DOI: 10.1093/jac/dkx327.

Clermont, O., Bonacorsi, S., and Bingen, E. (2000). Rapid and simple determination of *Escherichia coli* phylogenetic group. *Appl Environ Microbiol.* 66, 4555-4558. DOI: 10.1093/jac/dkp194.

Clermont, O., Lavollay, M., Vimont, S., Deschamps, C., Forestier, C., Branger, C., et al. (2008). The CTX-M-15-producing *Escherichia coli* diffusing clone belongs to a highly virulent B2 phylogenetic subgroup. *J. Antimicrob. Chemother.* 61, 1024-1028. DOI: 10.1093/jac/dkn084.

Clermont, O., Christenson, J.K., Denamur, E., and Gordon, D.M. (2013). The Clermont *Escherichia coli* phylo-typing method revisited: improvement of specificity and detection of new phylo-groups. *Environ. Microbiol. Rep.* 5, 58-65. DOI: 10.1111/1758-2229.12019.

Clermont, O., Dixit, O.V.A., Vangchhia, B., Condamine, B., Dion, S., Bridier-Nahmias, A., Denamur, E., Gordon, D. (2019). Characterization and rapid identification of phylogroup G in *Escherichia coli*, a lineage with high virulence and antibiotic resistance potential. *Environ Microbiol.* 21(8):3107-3117. DOI: 10.1111/1462-2920.14713.

Franklin, M.A., Francis, D.H., Baker, D., Mathew, A.G. (1996). A PCR-based method of detection and differentiation of K88+ adhesive *Escherichia coli*. *J. Vet. Diagn. Invest.* 8, 460-463. DOI: 10.1177/104063879600800410.

García-Meniño, I., García, V., Mora, A., Díaz-Jiménez, D., Flament-Simon, S.C., Alonso, M.P., Blanco, J.E., Blanco, M., Blanco, J. (2018). Swine enteric colibacillosis in Spain: pathogenic potential of *mcr-1* ST10 and ST131 *E. coli* isolates. *Front Microbiol* 9:2659. DOI: 10.3389/fmicb.2018.02659.

Gómez-Duarte, O.G., Arzuza, O., Urbina, D., Bai, J., Guerra, J., Montes, O., Puello, M., Me4ndoza, K., Castro, G.Y. (2010). Detection of *Escherichia coli* enteropathogens by multiplex polymerase chain reaction from children's diarrheal stools in two Caribbean-Colombian cities. *Foodborne Pathog Dis* 7:199–206. DOI: 10.1089/fpd.2009.0355.

Imberechts, H., De Greve, H., Schlicker, C., Bouchet, H., Pohl, P., Charlier, G., Bertschinger, H., Wild, P., Vandekerckhove, J., Van Damme, J., et al. (1992). Characterization of F107 fimbriae of *Escherichia coli* 107/86, which causes edema disease in pigs, and nucleotide sequence of the F107 major fimbrial subunit gene, *fedA*. *Infect. Immun.* 60, 1963-1971. DOI: 10.1128/iai.60.5.1963-1971.1992.

Lescat, M., Clermont, O., Woerther, P.L., Glodt, J., Dion, S., Skurnik, D., *et al*. (2013). Commensal *Escherichia coli* strains in Guiana reveal a high genetic diversity with host-dependant population structure. *Environ Microbiol Rep*. 5, 49-57. DOI: 10.1111/j.1758-2229.2012.00374.x.

Mora, A., Herrrera, A., Lopez, C., Dahbi, G., Mamani, R., Pita, J.M., Alonso, M.P., Llovo, J., Bernardez, M.I., Blanco, J.E., Blanco, M., Blanco, J. (2011). Characteristics of the Shiga-toxin-producing enteroaggregative *Escherichia coli* O104:H4 German outbreak strain and of STEC strains isolated in Spain. *Int. Microbiol.* 14, 121-141. DOI: 10.2436/20.1501.01.142.

Mora, A., Viso, S., López, C., Alonso, M.P., García-Garrote, F., Dabhi, G., Mamani, R., Herrera, A., Marzoa, J., Blanco, M., Blanco, J.E., Moulin-Schouleur, M., Schouler, C., Blanco, J. (2013). Poultry as reservoir for extraintestinal pathogenic *Escherichia coli* O45:K1:H7-B2-ST95 in humans. *Vet. Microbiol.* 167, 506-512. DOI: 10.1016/j.vetmic.2013.08.007.

Penteado, A.S., Ugrinovich, L.A., Blanco, J., Blanco, M., Blanco, J.E., Mora, A., Andrade, J.R., Correa, S.S., Pestana de Castro, A.F. (2002). Serobiotypes and virulence genes of *Escherichia coli* strains isolated from diarrheic and healthy rabbits in Brazil. *Vet. Microbiol.* 89, 41-51. DOI: 10.1016/s0378-1135(02)00148-7.

Rebelo, A.R., Bortolaia, V., Kjeldgaard, J.S., Pedersen, S.K., Leekitcharoenphon, P., Hansen, I.M., Guerra, B., Malorny, B., Borowiak, M., Hammerl, J.A., Battisti, A., Franco, A., Alba, P., Perrin-Guyomard, A., Granier, S.A., De Frutos Escobar, C., Malhotra-Kumar, S., Villa, L., Carattoli, A., Hendriksen, R.S. (2018). Multiplex PCR for detection of plasmid-mediated colistin resistance determinants, *mcr-1*, *mcr-2*, *mcr-3*, *mcr-4* and *mcr-5* for surveillance purposes. *Euro Surveill*. 23(6):17-00672. DOI: 10.2807/1560-7917.ES.2018.23.6.17-00672.

Saladin, M., Cao, V.T., Lambert, T., Donay, J.L., Herrmann, J.L., Ould-Hocine, Z., Verdet, C., Delisle, F., Philippon, A., Arlet, G. (2002). Diversity of CTX-M beta-lactamases and their promoter regions from Enterobacteriaceae isolated in three Parisian hospitals. *FEMS Microbiol. Lett.* 209, 161-168. DOI: 10.1111/j.1574-6968.2002.tb11126.x.

Scheutz, F., Teel, L.D., Beutin, L., Pierard, D., Buvens, G., Karch, H., Mellmann, A., Caprioli, A., Tozzoli, R., Morabito, S., Strockbine, N.A., Melton-Celsa, A.R., Sanchez, M., Persson, S., O'Brien, A.D. (2012). Multicenter evaluation of a sequence-based protocol for subtyping Shiga toxins and standardizing Stx nomenclature. *J. Clin. Microbiol.* 50, 2951-2963.

Schultsz, C., Pool, G.J., van Ketel, R., de Wever, B., Speelman, P., Dankert, J. (1994). Detection of enterotoxigenic *Escherichia coli* in stool samples by using nonradioactively labeled oligonucleotide DNA probes and PCR. *J. Clin. Microbiol.* 32, 2393-2397. DOI: 10.1128/jcm.32.10.2393-2397.1994.

Simarro, E., Navarro, F., Ruiz, J., Miró, E., Gómez, J., Mirelis, B. (2000). *Salmonella enterica* serovar virchow with CTX-M-like beta-lactamase in Spain. *J. Clin. Microbiol.* 38, 4676-4678. DOI: 10.1128/jcm.38.12.4676-4678.2000.
