# Supplementary figures and images for: Mobile colistin resistance (MCR), extended-spectrum beta-lactamase (ESBL) and multidrug resistance monitoring in Escherichia coli (commensal and pathogenic) in pig farming: need of harmonized guidelines and clinical breakpoints
Source: Front Microbiol. 2022 Dec 2;13:1042612. doi: 10.3389/fmicb.2022.1042612 (PMC9756432; doi:10.3389/fmicb.2022.1042612)

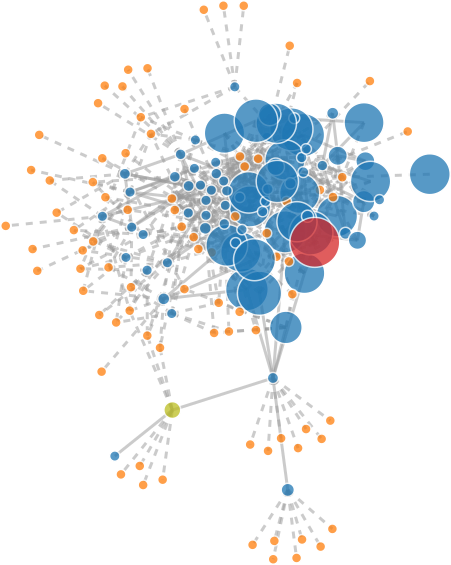

Supplement: Supplementary file 3 [file Image_1.jpg]
